# Older adult-onset Alexander disease with atypical clinicoradiological features: a case report

**DOI:** 10.3389/fneur.2023.1139047

**Published:** 2023-06-15

**Authors:** You-Ri Kang, Tai-Seung Nam, Jae-Myung Kim, Kyung Wook Kang, Seung-Han Lee, Seong-Min Choi, Myeong-Kyu Kim

**Affiliations:** ^1^Department of Neurology, Chonnam National University Hospital, Gwangju, Republic of Korea; ^2^Department of Neurology, Chonnam National University Medical School, Gwangju, Republic of Korea

**Keywords:** age of onset, Alexander disease, brainstem atrophy, magnetic resonance imaging, medulla oblongata

## Abstract

Alexander disease (AxD) is a rare autosomal dominant astrogliopathy caused by mutations in the gene encoding for glial fibrillary acidic protein. AxD is divided into two clinical subtypes: type I and type II AxD. Type II AxD usually manifests bulbospinal symptoms and occurs in the second decade of life or later, and its radiologic features include tadpole-like appearance of the brainstem, ventricular garlands, and pial signal changes along the brainstem. Recently, eye-spot signs in the anterior medulla oblongata (MO) have been reported in patients with elderly-onset AxD. In this case, an 82-year-old woman presented with mild gait disturbance and urinary incontinence without bulbar symptoms. The patient died 3 years after symptom onset as a result of rapid neurological deterioration after a minor head injury. MRI showed signal abnormalities resembling angel wings in the middle portion of the MO along with hydromyelia of the cervicomedullary junction. Herein, we report the case of this patient with older adult-onset AxD with an atypical clinical course and distinctive MRI findings.

## Introduction

Alexander disease (AxD) is an inherited progressive neurodegenerative disease caused by a mutation in the gene encoding for glial fibrillary acidic protein (GFAP). AxD has traditionally been classified into three types based on age at onset (AAO): infantile-onset (from birth to 2 years), juvenile-onset (2–14 years), and adult-onset (>14 years) (1). However, another classification system based on statistical analyses of 215 AxD cases was proposed in 2011 (2). According to this system, type I AxD manifests cerebral symptoms and signs occurring before the age of 4 years and cerebral white matter (WM) abnormalities with frontal predominance, while type II usually manifests bulbospinal symptoms in the second decade of life or later and posterior fossa WM abnormalities (2). A tadpole-like form of brainstem atrophy is the most typical manifestation of type II AxD; this can be caused by significant atrophy of the medulla oblongata (MO) and upper cervical spinal cord (3). Other radiologic findings include pial fluid-attenuated inversion recovery (FLAIR) signal changes in the brainstem (4), ventricular garlands (5), and the “eye-spot” sign in the anterior portion of the MO (6).

We managed a patient with older adult-onset AxD who presented with mild gait disturbance and urinary incontinence and without bulbar symptoms. The patient died 3 years after symptom onset as a result of post-traumatic neurological deterioration. MRI showed signal abnormality in the middle portion of the MO and hydromyelia of the cervicomedullary junction (CMJ), findings that have not been previously described. Herein, we report the case of this patient with type II AxD with an atypical clinical course and distinct MRI findings.

## Case description

**Figure 1:**
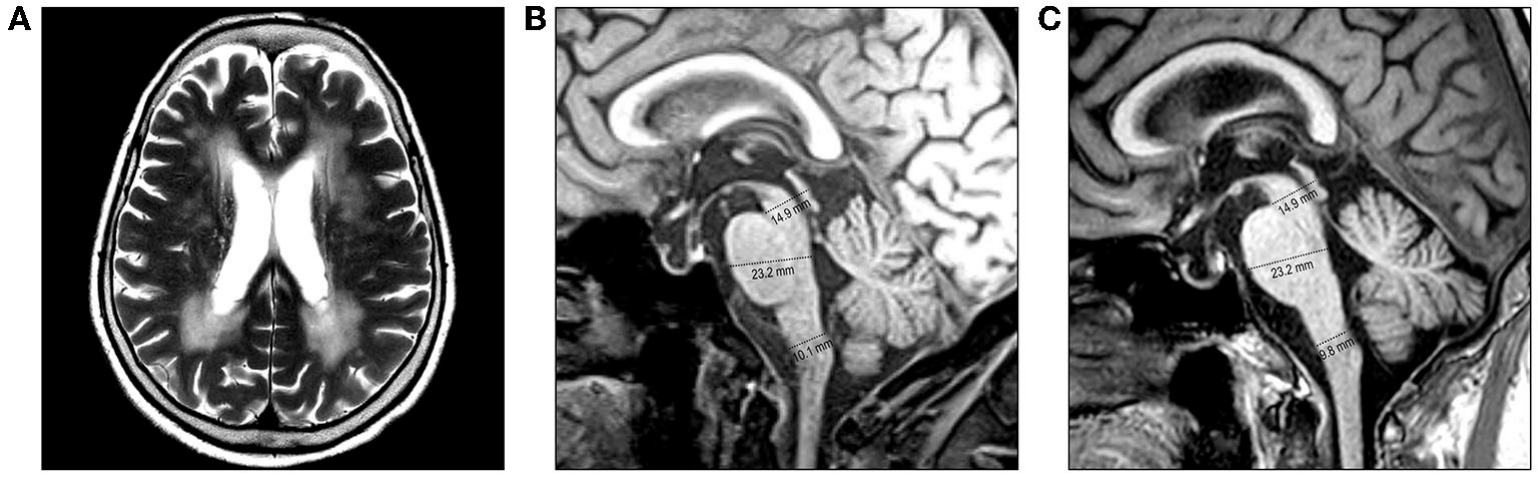
Brain MRI of the patient. **(A, B)** MRI performed at the initial examination. **(A)** Axial T2-weighted image reveals periventricular and deep white matter hyperintensities. **(B)** Diameters of the midbrain, pons, and medulla oblongata (MO) are 14.9 mm, 23.3 mm, and 10.1 mm, respectively, on the midsagittal T1-weighted image. **(C)** MRI performed after a minor head injury shows no prominent changes. The diameters of the brainstem sections were measured as the anteroposterior distance according to the method proposed by Yoshida et al. (7).

**Figure 2:**
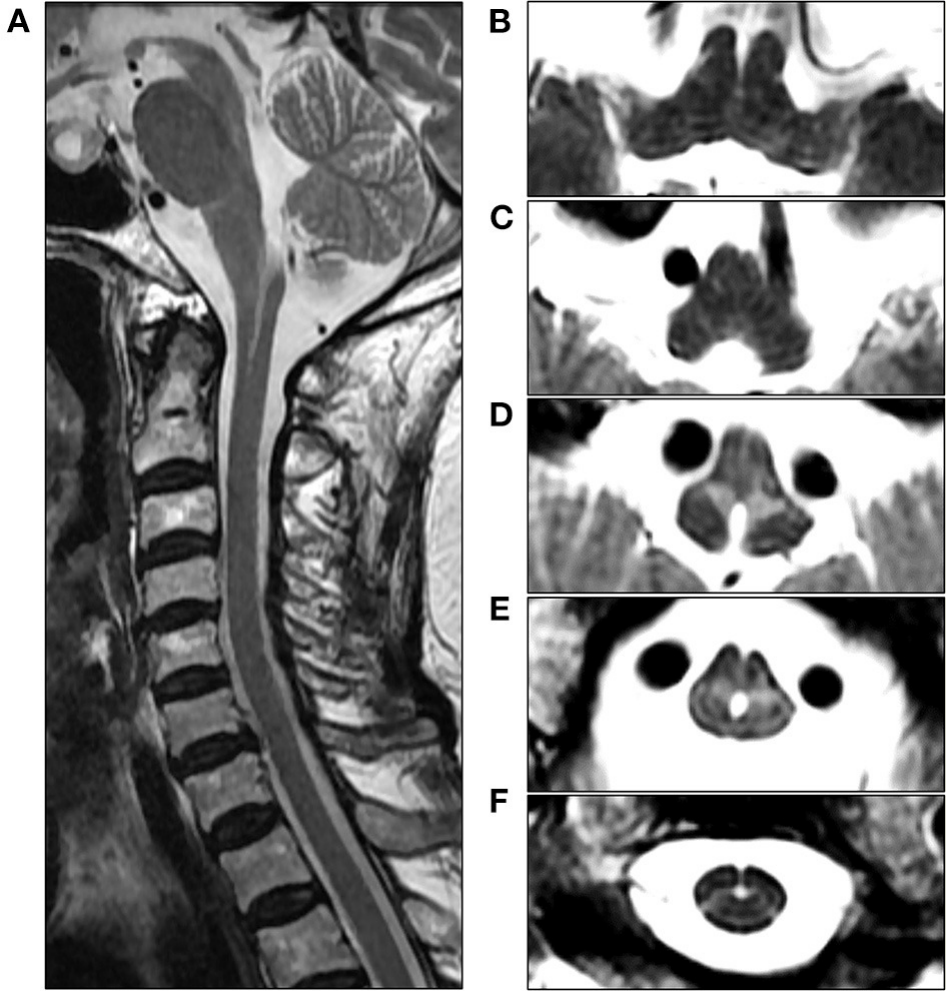
Cervical spine MRI of the patient at the initial examination. **(A)** Sagittal T2-weighted image reveals unevenly shaped hydromyelia at the cervicomedullary junction (CMJ). **(B, C)** Axial T2-weighted images at the level of the rostral medulla oblongata (MO) show normal findings in the absence of signal abnormalities in the pyramids. **(D)** Axial T2-weighted image at the level of the caudal MO reveals bilaterally symmetric hyperintensities around the central canal, resembling angel wings. **(E)** Axial T2-weighted image at the level of the CMJ reveals a hydromyelia and signal abnormalities radiating laterally from the central canal. **(F)** Axial T2-weighted image at the C2 vertebral level shows the cervical cord with normal appearance.

An 82-year-old woman with diabetes mellitus presented with a several-month history of unsteady gait and urinary incontinence. Neurological examination showed mild spasticity in the lower limbs and generalized hyperreflexia, but no Babinski sign or ankle clonus was present. We observed no bulbar symptoms or signs, including dysarthria, dysphagia, or dysphonia, and the results of tests for extrapyramidal symptoms, ocular movements, and parkinsonism were unremarkable. Her mini-mental state examination score was 25/30. A urodynamic study revealed dysfunction during the storage phases, suggesting neurogenic detrusor overactivity. Normal pressure hydrocephalus was initially suspected based on the triad of symptoms of gait disturbance, cognitive impairment, and urinary incontinence. Brain MRI showed periventricular and deep WM signal changes in the FLAIR image with no ventricular enlargement (Evans index = 0.26) (Figure 1A). Electrodiagnostic studies, including a nerve conduction study, needle electromyography, and evoked potentials, were conducted to evaluate amyotrophic lateral sclerosis or cervical spondylotic myelopathy, but these showed unremarkable results except for prolonged central motor conduction time to the upper and lower limbs in motor-evoked potentials (MEPs). In the spine MRI, mild atrophy of the MO was suspected; the ratio of the sagittal diameter of the MO to that of the pons was 0.43 (Figure 1B). No pial FLAIR signal changes were observed, nor was the eye-spot sign in the anterior MO. Intriguingly, hydromyelia in the CMJ and signal abnormalities radiating laterally from the central canal in the middle portion of the caudal MO were observed (Figure 2). GFAP gene sequencing revealed a heterozygous missense mutation (c.197G > A, p.Arg66Gln) previously reported to be pathogenic (2), and the patient was ultimately diagnosed with type II AxD. Although mild gait unsteadiness persisted, the patient could walk independently without assistance for approximately 2 years after the diagnosis. However, after a minor head injury resulting from a slip and fall, the patient became unable to walk, and dysarthria and dysphagia developed. Follow-up brain MRI showed no evidence of intracranial hemorrhage. There were no notable changes in the diameters of the sections of the brainstem, although the diameter of the MO was slightly decreased (Figure 1C). Eventually, the patient died of recurrent episodes of pneumonia 3 years after symptom onset.

## Discussion

Type II AxD usually manifests bulbospinal symptoms and signs in the second decade of life or later (2). In our patient, neurological symptoms developed after the age of 80 years and remained mild, without apparent progression until the patient experienced head trauma, which is consistent with the previous speculation that advanced age of onset is associated with a milder clinical course (2). Conversely, our patient died 3 years after symptom onset, which is in line with a previous report that patients with older adult-onset AxD aged >65 years may experience more rapid disease progression than those with younger adult-onset AxD and become dependent within 2 years of onset (8). Even a minor head injury has been reported to cause acute neurological deterioration in AxD (9), which is similar to the case of our patient who died within 8 months of post-traumatic neurological exacerbation. The association between AAO and disease course in adult-onset AxD remains unclear and inconclusive. However, our case of older adult-onset AxD highlights the fact that extrinsic factors, including head trauma, physical immobility, or infections, may accelerate disease progression or contribute to poor prognosis.

Among the various MRI features observed in later-onset AxD, the most typical finding is brainstem and spinal cord atrophy (3). In our case, the ratio of the sagittal diameter of the MO to that of the pons was slightly decreased, which is consistent with one of the MRI parameters suggested for distinguishing adult-onset AxD from other neurological disorders (7). However, tadpole-like brainstem atrophy was not distinctly evident. The most distinctive MRI findings in our case were the signal abnormalities resembling “angel wings” radiating from the central canal (Figures 2D, E) and hydromyelia of the CMJ, which have not yet been reported. Recently, a signal abnormality in the anterior portion of MO, referred to as the “eye-spot sign,” has been reported in elderly-onset AxD with mild MO atrophy and has been speculated to reflect myelin loss in the bilateral pyramids (6). In this case, our patient exhibited the pyramidal signs and MEP abnormalities, and thus the angel-wings-like signal change may be understood as being caused by the dysmyelination of the corticospinal fibers in the section where pyramidal tracts on both sides enter the spinal cord immediately after pyramidal decussation. However, this signal abnormality is located not in the medullary pyramids but in the middle portion of the MO, which suggests that it could not be simply explained by the hypothesis proposed by Yoshida et al. (6). Furthermore, the contiguity of signal change and hydromyelia at the caudal MO may indicate the contribution of the central canal to this distinctive MRI finding. The central canal is lined by the ependymal cell layer, surrounded by subependymal regions comprised of glial cells (10). Intriguingly, in an autopsy report of an 85-year-old AxD patient with no focal neurological deficit, the intense gliosis accompanied by abundant Rosenthal fibers was limited to the subependymal regions of the central canal, third ventricle, and fourth ventricle in the absence of macroscopic brainstem atrophy. Accordingly, the angel-wings-like signal change might reflect gliosis extending from the subependymal area of the central canal deep into the medullary parenchyma (11). Moreover, hydromyelia might be explained by the passive widening of the central canal secondary to the regional neurodegeneration of the subependymal area. Unfortunately, our hypotheses could not be substantiated since a postmortem examination was not performed in this case.

In summary, our case highlights several interesting characteristics of older adult-onset type II AxD, including mild spastic gait and urinary incontinence in the absence of bulbar symptoms, with rapid disease progression after a minor head injury. Radiologically, angel-wings-like signal abnormalities along with hydromyelia at the level of CMJ may be another feature of type II AxD. This case helps us understand the clinical and radiologic features of older adult-onset AxD.

## Data availability statement

The datasets presented in this article are not readily available because of ethical and privacy restrictions. Requests to access the datasets should be directed to the corresponding author.

## Ethics statement

The studies involving human participants were reviewed and approved by Institutional Review Board at Chonnam National University Hospital. The patients/participants provided their written informed consent to participate in this study. Written informed consent was obtained from the individual(s) for the publication of any potentially identifiable images or data included in this article.

## Author contributions

Y-RK: data curation and writing of original draft. T-SN: conceptualization, writing (review and editing), and supervision. J-MK, KK, and S-MC: formal analysis. S-HL: data curation and formal analysis. M-KK: formal analysis and supervision. All authors contributed to the article and approved the submitted version.
